# Use of α-cyclodextrin to Promote Clean and Environmentally Friendly Disinfection of Phenolic Substrates via Chlorine Dioxide Treatment

**DOI:** 10.3389/fchem.2020.00641

**Published:** 2020-07-31

**Authors:** Sauradip Chaudhuri, Dana J. DiScenza, Thomas B. Boving, Alan Burke, Mindy Levine

**Affiliations:** ^1^McGovern Medical School, University of Texas Health Science Center at Houston, Houston, TX, United States; ^2^Department of Chemistry, University of Virginia, Charlottesville, VA, United States; ^3^Department of Geosciences/Department of Civil and Environmental Engineering, University of Rhode Island, Kingston, RI, United States; ^4^Independent Researcher, North Kingstown, RI, United States; ^5^Department of Chemical Sciences, Ariel University, Ariel, Israel

**Keywords:** cyclodextrin, bisphenol (BPA), 2-phenylphenol, hydrophobic encapsulation, chlorination

## Abstract

The use of chlorine dioxide to disinfect drinking water and ameliorate toxic components of wastewater has significant advantages in terms of providing safe water. Nonetheless, significant drawbacks toward such usage remain. These drawbacks include the fact that toxic byproducts of the disinfection agents are often formed, and the complete removal of such agents can be challenging. Reported herein is one approach to solving this problem: the use of α-cyclodextrin to affect the product distribution in chlorine dioxide-mediated decomposition of organic pollutants. The presence of α-cyclodextrin leads to markedly more oxidation and less aromatic chlorination, in a manner that is highly dependent on analyte structure and other reaction conditions. Mechanistic hypotheses are advanced to explain the cyclodextrin effect, and the potential for use of α-cyclodextrin for practical wastewater treatment is also discussed.

**Graphical Abstract:**
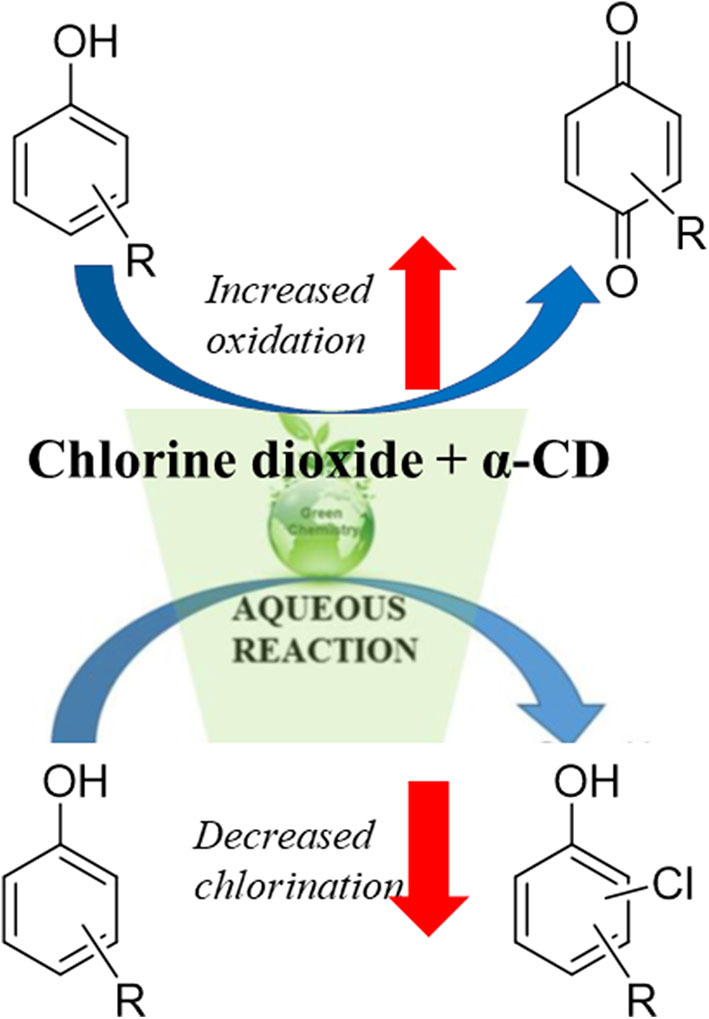
Chlorine dioxide-mediated decontamination of aromatic pollutants in water in the presence of α-cyclodextrin (α-CD) changes the reaction pathway, leading to decreased chlorination, and increased oxidation.

## Introduction

The decontamination of the water supply from a variety of organic pollutants (Cravotto et al., 2005), including phthalates (Przybylinska and Wyszkowski, 2016), biphenyls (Benoit et al., 2016), and bisphenol derivatives (Onundi et al., 2017) is an important challenge with a variety of industrial and public health applications (Foo and Hameed, 2010; Shah et al., 2016). Methods to achieve such decontamination to facilitate access to clean drinking water tend to rely on the application of large quantities of disinfectants, oxidants, or decomposition reagents (Kim et al., 1999), with newer methods including the use of photochemical (Laxma Reddy et al., 2017), electrochemical (Oturan et al., 2009), and sonochemical (Joseph et al., 2009) decontamination procedures. While these methods are effective in reducing the quantities of known organic pollutants (Besner et al., 2008), the decomposition products of both the pollutants and of chemicals used for disinfection have not been well-characterized (Wang et al., 2014), and methods to remove such decomposition products from the water stream are poorly developed. This is particularly concerning because many of these decomposition products are likely to have similar or even worse toxicities compared to their associated starting materials (Li and Mitch, 2018).

One popular disinfection agent is chlorine dioxide, which has been used for the decontamination of wastewater from pathogens (Banach et al., 2015), as part of the seawater desalination process (Kim et al., 2015), and for the removal of antibiotics and other pharmaceuticals from drinking water (Dodd, 2012). Despite the widespread usage of chlorine dioxide, concerns remain about its toxicity (Ma et al., 2017), and about the toxicity of pollutant byproducts that result from chlorine dioxide treatment (Colman et al., 2011). Efforts to mitigate this toxicity have focused on alternative disinfection treatments (Meireles et al., 2016), on immobilization of chlorine dioxide to minimize the diffusion of toxic byproducts (He et al., 2014), and on the combined use of chlorine dioxide and other water treatments (Hsu and Huang, 2015). The use of supramolecular constructs and/or adducts of chlorine dioxide as strategies for mitigating chlorine dioxide-induced water treatment toxicity has not been reported to date, despite the fact that chlorine dioxide is known to form a variety of supramolecular adducts (Loginova et al., 2011; Palcso et al., 2019), including with α-cyclodextrin (Wambaugh et al., 2013). Moreover, supramolecular association with common organic pollutants is well-known, including cyclodextrin complexation with the classes of pollutants mentioned above [phthalates (Cromwell et al., 2019), biphenyls (Serio et al., 2013), and bisphenols (DiScenza et al., 2018)]. Finally, supramolecular complexation in general (Chang et al., 2017), and cyclodextrin complexation in particular (Aiassa et al., 2016), has been shown to result in significantly altered and often reduced toxicities, which provides another potential avenue by which toxicity of the water stream can be mitigated.

In general, cyclodextrin complexation has been shown to rely heavily on hydrophobic association of hydrophobic small molecules inside the hydrophobic interior cavity of the cyclodextrin hosts. Such host-guest complexes have a strong dependence on the steric complementarity between the cavity size and the size of the guest, with single aromatic ring compounds reported to bind strongly in α-cyclodextrin (Connors and Pendergast, 1984; Pendergast and Connors, 1985) and larger aromatic (and hydrophobic aliphatic compounds) reported to bind in β-cyclodextrin (Celebioglu et al., 2019; Yu et al., 2019). Of note, moving to the even larger γ-cyclodextrin oftentimes results in the formation of ternary complexes, where two small molecule guests bind simultaneously inside the larger γ-cyclodextrin core (Hamai, 2010; Saokham et al., 2018). For the most common analytes involved in aqueous contamination (*vide infra*), the single aromatic rings of these analytes indicate that they are likely to bind strongly in the α-cyclodextrin cavity. Such strong and sterically matched binding, in turn, is expected to affect the reactivity of these substrates and the distribution of products obtained, an expectation that was effectively borne out by the results of our experiments (*vide infra*). The use of larger cyclodextrins, by contrast, would lead to the formation of less sterically matched complexes, which would in turn impart lower selectivities and lower overall cyclodextrin-induced effects.

Recent reports from our research groups have focused on the design, optimization, and sensing applications of cyclodextrin complexes (for the Levine group) (Serio et al., 2015; Chaudhuri et al., 2018; Haynes et al., 2019), and on the engineering, deployment, and evaluation of water purification filters (for the Boving group) (Schifman et al., 2016; Eberle et al., 2017; Blanford et al., 2018), which combined have provided us with unique insight into the potential of cyclodextrins to benefit the water purification process. Reported herein are the results of our investigations into the effect of α-cyclodextrin complexation on chlorine dioxide-based water treatment, and how such complexation affects the quantity and distribution of degradation byproducts. Mechanistic insight into the specific role of α-cyclodextrin is also discussed.

## Experimental Section

### Materials and Methods

^1^H NMR experiments were conducted using a 400 MHz Bruker Avance spectrometer with D_2_O as a solvent. GC-MS analyses of reaction mixtures were carried out using a Shimadzu GCMS-QP2020 instrument. All chemicals were purchased from Sigma Aldrich chemical company or from Fisher Scientific and were used as received, without further purification.

### Method for the Preparation of Chlorine Dioxide Solution

An aqueous chlorine dioxide suspension was generated from the treatment of a solution of NaClO_2_ (ERCOPure™ 7.5) with activated HCl. A typical generation procedure involved the addition of 4 mL of 30–36% HCl to a mixture of 17.5 mL of ADOX™ 7.5 and 200 mL of deionized water. The reaction mixture was kept in a dark Amber bottle for ~24 h at room temperature. Prior to usage, the reaction mixture was further diluted to render a final chlorine dioxide concentration of ~1,095 ppm (confirmed via hand-held colorimetry using a Hach Digital Titrator).

### General Method for the Complexation of Chlorine Dioxide With α-cyclodextrin

The complexation of chlorine dioxide with α-cyclodextrin was obtained by mixing a solution of 6.2 mL of 1,100 ppm of ClO_2_ (100 μmol) with solid α-cyclodextrin (584 mg; 600 μmol) for 15–20 min.

### General Method for Determining the Reaction Progression

**Figure 1:**
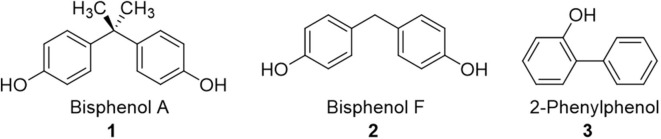
Structures of common aqueous pollutants investigated herein as substrates for chlorine dioxide-mediated degradation.

The reaction mixture containing the organic analyte (compounds **1-3**, Figure 1) was treated with ClO_2_ and allowed to react at the specified temperature for a certain amount of time, after which time the mixture was treated with concentrated sodium sulfite (Na_2_SO_3_) solution to quench the excess chlorine dioxide. The resulting solution was extracted with ethyl acetate. An aliquot of the organic phase was injected into the GC-MS for analysis, which enabled us to identify unreacted starting material, as well as new peaks corresponding to the formation of a variety of oxidation and chlorination products.

### General Method for Measuring the Binding of Analytes in α-cyclodextrin

Binding of analytes with α-cyclodextrin was investigated via ^1^H NMR titrations (Roselet and Kumari, 2017). A mixture of analytes (20 μmol) with α-cyclodextrin (0.0–5.0 equivalents) in D_2_O were investigated via ^1^H NMR spectroscopy, and the resulting shifts in the positions of the NMR signals were used to confirm supramolecular complexation.

## Results and Discussion

### Analyte Selection

There are a broad variety of organic pollutants that contaminate water supplies, including phthalates, biphenyls, and bisphenol derivatives (*vide supra*). We have selected three common pollutants to focus on in this paper, all of which have been reported to interact with α-cyclodextrin: bisphenol A (BPA) (analyte **1**) (Araki et al., 2001), bisphenol F (BPF) (analyte **2**) (Xiao et al., 2007), and 2-phenylphenol (analyte **3**) (Burkert et al., 1981), with the expectation that supramolecular interactions of the pollutants with α-cyclodextrin is likely to affect their chlorine dioxide-mediated degradation. Moreover, the selection of three pollutants with similar structures is expected to provide important insight into the structural selectivity of α-cyclodextrin complexation, and how such selectivity affects the chlorine dioxide-mediated degradation processes. Finally, the inclusion of BPF in addition to BPA is important, as BPA derivatives such as BPF are increasingly used as commercially available substitutes for BPA (Bjornsdotter et al., 2017; Wu et al., 2018), with evidence indicating analogous or even worse toxicity compared to BPA (den Braver-Sewradj et al., 2020).

### Initial Screening

**Figure 2:**
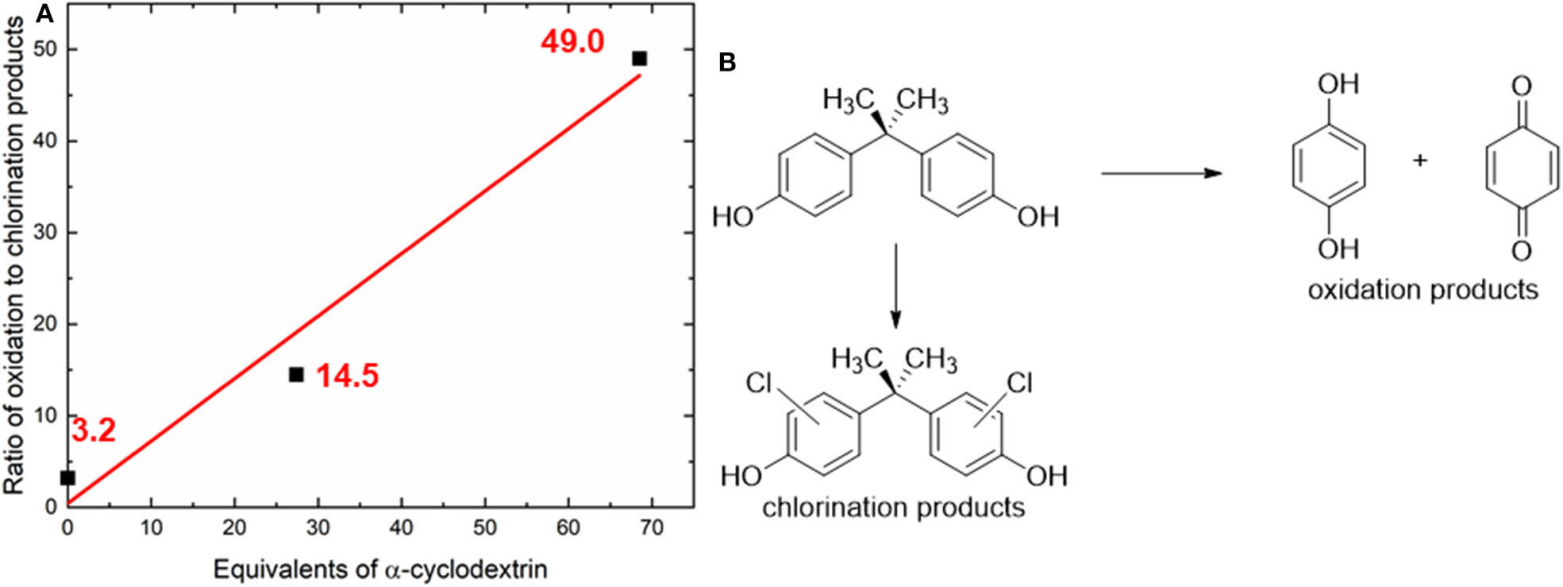
**(A)** Illustration of how the addition of α-cyclodextrin dramatically increases the oxidation to chlorination ratio in bisphenol A degradation at room temperature. **(B)** Illustration of oxidation and chlorination products that are produced from the chlorine dioxide-mediated decomposition of bisphenol A. Only one set of experiments was conducted to screen the reaction temperature; after determining that significant amounts of starting material remained; additional experimental variations were pursued. The red line represents the best linear fit to the data; Equation: *y* = 0.6821x +_0.4289; *R*^2^ = 0.9715.

Initial screening of reaction conditions started with room temperature treatment of analyte **1** with chlorine dioxide and α-cyclodextrin (0–60 equivalents relative to the substrate). BPA was found to undergo decomposition into single aromatic ring oxidized units (quinols and quinones), as well as undergo chlorination on residual starting material to form chlorinated BPA analogs. Under these conditions, increasing the equivalents of cyclodextrin led to a dramatic increase in the ratio of oxidation products to chlorination products, from a ratio of 3.2 without any cyclodextrin to a ratio of oxidation to chlorination of 49 measured at the highest concentration of α-cyclodextrin (Figure 2).

**Figure 3:**
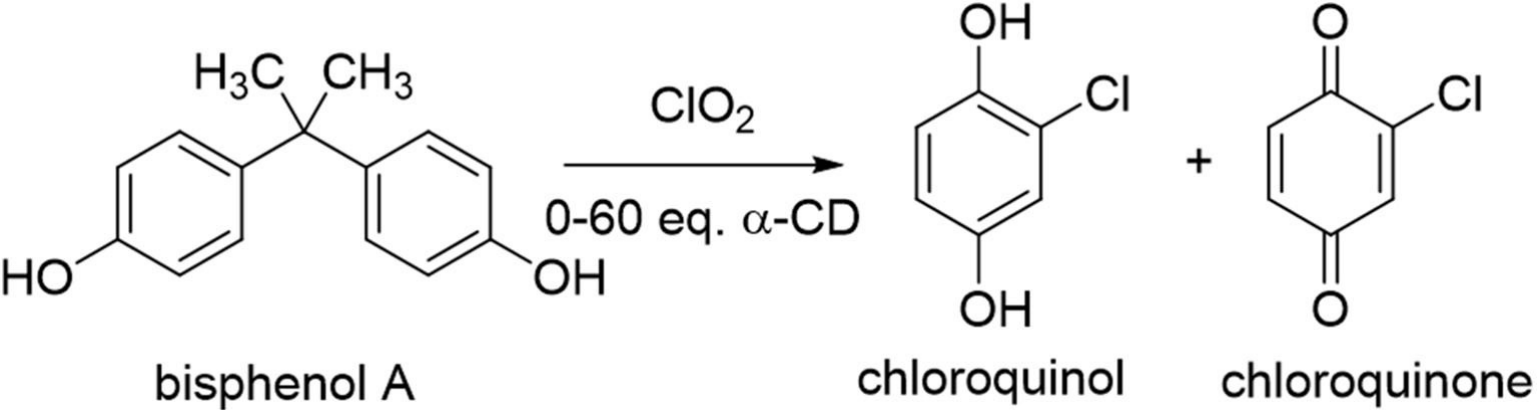
Illustration of the degradation reaction of bisphenol A upon treatment with chlorine dioxide at 40°C.

However, even after 24 h, significant unreacted starting material remained (up to 72%). Raising the reaction temperature slightly, to 40°C, resulted in complete consumption of the starting material but dramatic changes in the reaction products, with nearly exclusive formation of chlorinated oxidation products chloroquinol and chloroquinone (Figure 3). This significant change in consumption of the starting material with only a mild increase in the temperature of the reaction is likely due to increased reaction kinetics at the elevated temperature. The stability of the cyclodextrin complexes is likely also affected by the increased reaction temperature, which in turn leads to changes in the distribution of products observed. Increasing the equivalents of α-cyclodextrin in this system led to moderate increases in the ratio of chloroquinol to chloroquinone obtained, with overall limited changes in the overall oxidation products obtained (Table 1). This could likely be due to the stronger binding of quinols to α-cyclodextrin, thereby inhibiting their subsequent oxidation into quinones.

**Table 1:** Effects of α-cyclodextrin addition on the distribution of decomposition products obtained from chlorine dioxide treatment of bisphenol A^a^.

**Equivalents of α-cyclodextrin**	**Ratio of chloroquinol to chloroquinone**	**Overall oxidation product %**
0	1.9	56.2%
27.4	3.5	53.6%
68.5	4.3	55.3%

a *Reactions were run at 40°C for 24 h*.

**Figure 4:**
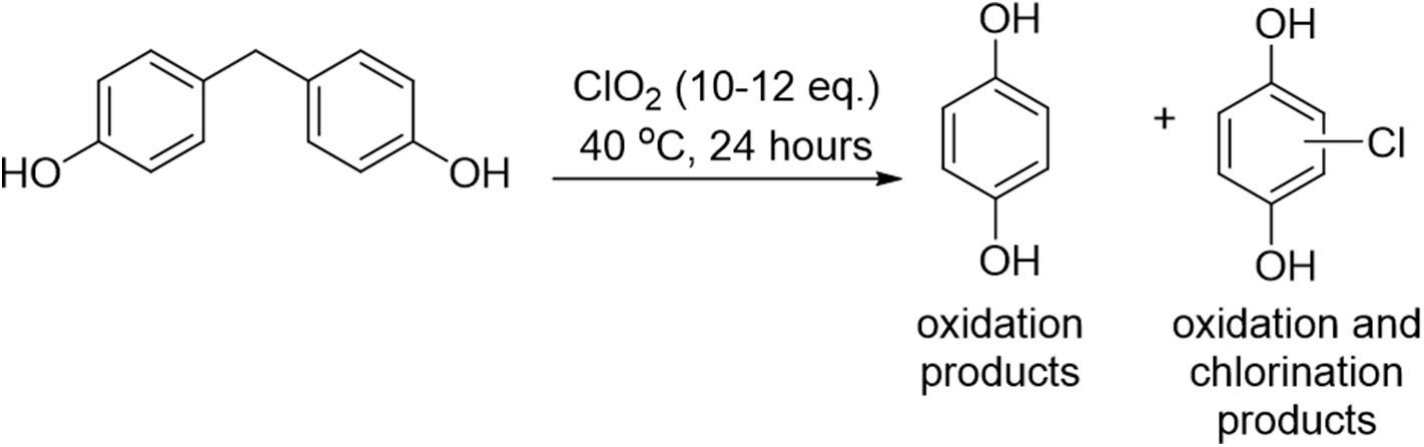
Illustration of how the chlorine dioxide-mediated decomposition of bisphenol F leads to the formation of quinols and chloroquinols exclusively.

In contrast to the results obtained for analyte **1**, treatment of bisphenol F (analyte **2**) with chlorine dioxide at 40°C led to 100% oxidation products, with both unsubstituted quinols and chloroquinols formed (Figure 4). The ratio of oxidation products (100% of the product mixture) to chlorination products (namely, the formation of chloroquinols) was calculated, and the results summarized in Table 2. Of note, substantial increases in the ratio of quinol to chloroquinol with increasing concentration of cyclodextrin indicates a decrease in the chlorination byproducts, which strongly suggests that cyclodextrin complexation plays a role in inhibiting that reaction pathway (*vide infra*).

**Table 2:** Ratio of oxidation to chlorination products formed by treating aromatic analytes **2** and **3** with chlorine dioxide with varying cyclodextrin equivalents^a^.

**Analyte**	**0 eq. α-CD**	**24 eq. α-CD**	**60 eq. α-CD**
**2**	1.2	1.5	1.5
**3**	3.2	4.5	5.1

a *Reactions were run at 40°C for 24 h, and the ratio of oxidation to chlorination products was determined via GC-MS analysis*.

**Figure 5:**
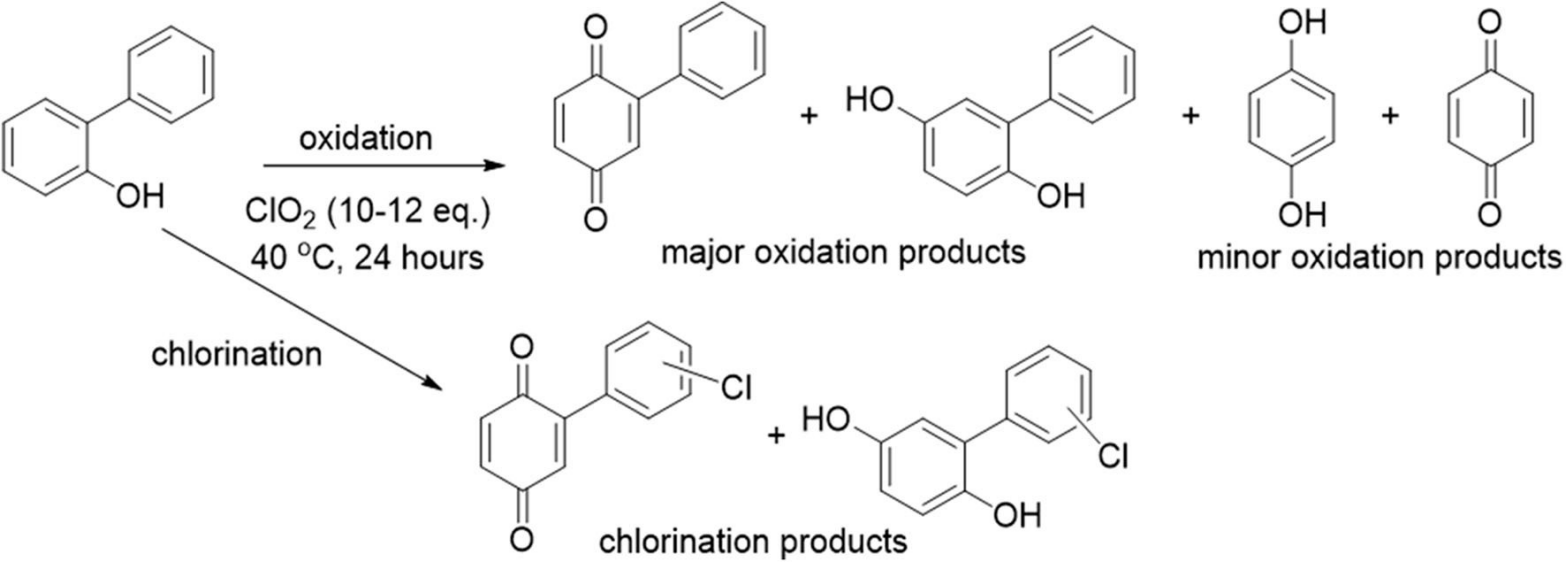
Illustration of the products formed from chlorine dioxide-mediated decomposition of analyte **3**.

In contrast to analytes **1** and **2**, analyte **3** (2-phenylphenol) underwent complete decomposition with ClO_2_ treatment, yielding a much more complex product profile. Unlike analytes **1** and **2**, which formed predominantly single aromatic ring oxidation products, the chlorine dioxide treatment of analyte **3** led to only minor amounts of such products, with the majority of oxidation products maintaining the core biphenyl structure. Such differences in product distribution between the analytes strongly suggests that the bridging methylene unit of the bisphenol structures of **1** and **2** (absent in analyte **3**) provided a site for C-C bond cleavage that enabled single aromatic ring products to form. In addition to the biphenyl-containing oxidation products, a variety of chlorinated products were also formed, most of which resulted from chlorination of the initially formed oxidized compounds (Figure 5). The ratio of oxidation products to chlorination products formed from chlorine dioxide-mediated decomposition of analyte **3**, with increasing equivalents of α-cyclodextrin effectively protecting the aromatic ring from undesired chlorination reactions (as shown by increasing values of the oxidation to chlorination ratio observed, Table 2).

Overall, the results obtained for analyte **1** at room temperature, and analytes **2** and **3** at 40°C indicate that the presence of cyclodextrin markedly increases the percentage of non-chlorinated oxidation products formed, with the one anomalous result, obtained for the chlorine dioxide treatment of analyte **1** at 40°C, discussed later in the manuscript. Of note, both substrates with bridging methylene units (compounds **1** and **2**) decomposed primarily into single aromatic ring oxidized units [(chloro)quinols and (chloro)quinones]. Such results have substantial relevance from a practical as well as a fundamental scientific perspective. From a practical perspective, chlorinated byproducts formed from chlorine dioxide mediated decomposition generally have higher reported toxicities than the non-chlorinated, oxidation products formed (Li and Mitch, 2018). As a result, the ability to decrease the relative amount of chlorinated products through α-cyclodextrin addition is particularly attractive, especially as α-cyclodextrin itself has almost no reported toxicity (Cal and Centkowska, 2008), and in fact has been used for a variety of biomedical applications due to its generally recognized safety (Szente et al., 2018). From a fundamental perspective, the fact that α-cyclodextrin complexation suppresses the chlorination processes is likely due to hydrophobic encapsulation of the phenyl rings in the cyclodextrin cavity, in a way that provides steric shielding and prevents aromatic chlorination from occurring (*vide infra*). Such supramolecular shielding provides insight into the mechanism of cyclodextrin complexation, how such complexation depends on the structure of the encapsulated guest, and how such complexes affect guest reactivity.

### Mechanistic Investigations

**Figure 6:**
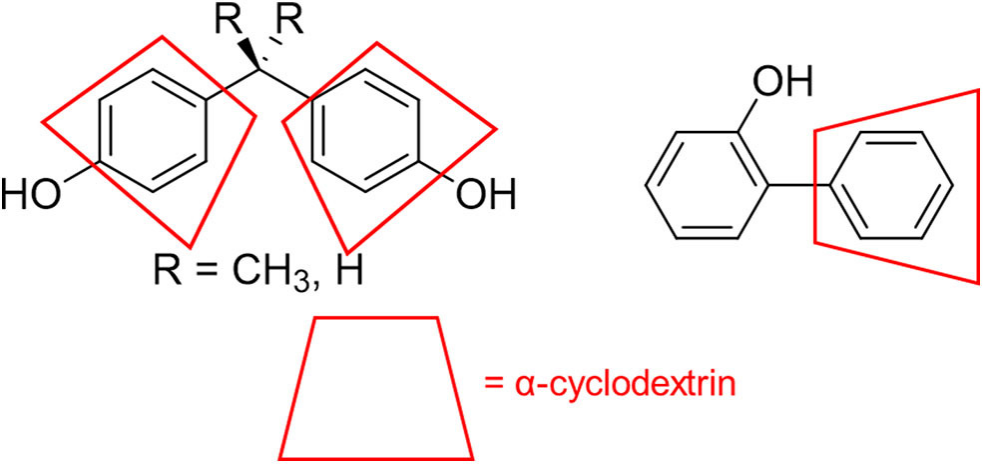
Schematic illustration of how α-cyclodextrin binds to aromatic rings on the pollutant structures to limit aromatic chlorination.

There are multiple fundamental mechanistic questions involved in this process, including how α-cyclodextrin affects the product distribution of chlorine dioxide mediated degradation, as well as how variations in substrate structure affect product distribution and the underlying reaction mechanism. Most likely, the binding of phenyl groups in the cyclodextrin cavity (Figure 6) provides supramolecular steric shielding from undesired aromatic chlorination. Moreover, the benzylic position on the substituent, which must be accessed to effect oxidation reactions, remains relatively unhindered. Electronic activation of that benzylic position through hydrogen bonding to the rims of cyclodextrins can also accelerate the desired oxidation reactions, and evidence for such activation is provided through ^1^H NMR analysis (*vide infra*).

Overall, the treatment of the analytes with chlorine dioxide led to highly analyte-specific results. Both analytes **1** and **2** yielded oxidation products with primarily single ring aromatic groups, whereas analyte **3** maintained its biphenyl structure, indicating that the bridging methylene group of analytes **1** and **2** plays a key role in facilitating oxidative bond cleavage. Differences in the product distribution of analytes **1** and **2** indicate a markedly more complex product mixture for analyte **1**'s treatment with chlorine dioxide, with major products of chloroquinol and chloroquinone and numerous minor products, generally with higher molecular weights (indicating radical-radical recombination). For analyte **1**, the fact that the ratio of chloroquinol to chloroquinone increased with increasing equivalents of α-cyclodextrin indicates that the cyclodextrin effectively inhibits oxidation of chloroquinol. In contrast, increasing the equivalents of α-cyclodextrin in the analyte **2** decomposition process led to an increased ratio of quinol to chloroquinol, which indicates that the chlorination reaction pathway was inhibited by α-cyclodextrin.

**Figure 7:**
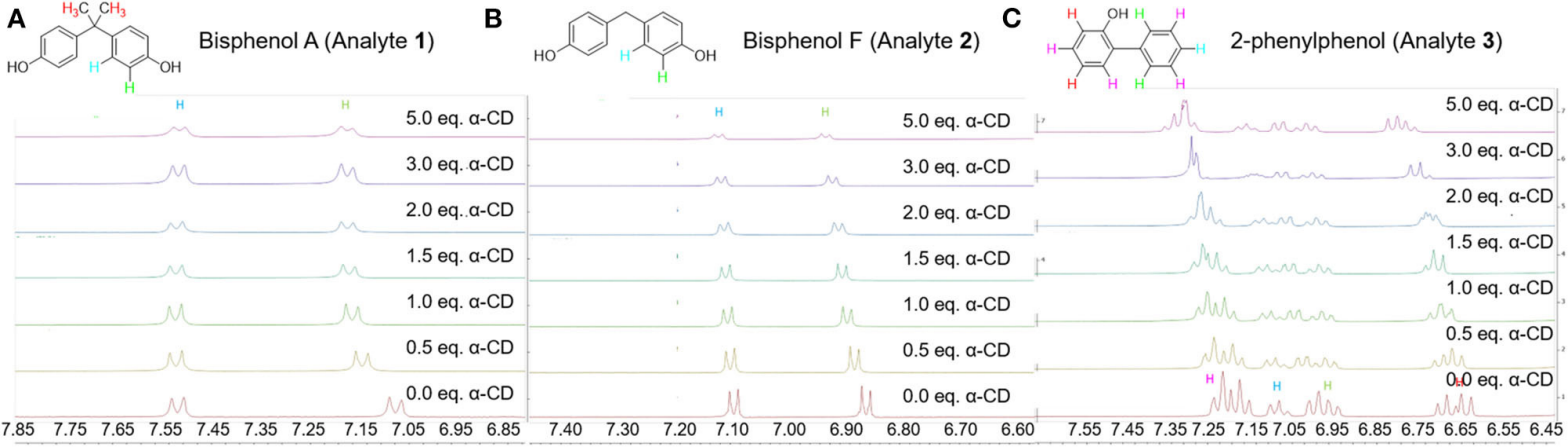
Copies of ^1^H NMR spectra of the aromatic protons of **(A)** analyte **1**; **(B)** analyte **2**; and **(C)** analyte **3** in the presence of increasing equivalents of α-cyclodextrin.

The proposed mechanism is further supported by ^1^H NMR chemical shift studies of the analytes in presence of increasing equivalents of α-cyclodextrin, and key results are summarized in Table 3 and Figure 7. In particular, all aromatic protons of the analytes demonstrated significant chemical shifts upon the addition of increasing concentrations of α-cyclodextrin, supporting supramolecular encapsulation of the type shown in Figure 6. Moreover, for analytes **1** and **2**, significant changes in chemical shift were also observed for the methyl group protons at the bridging carbon (for analyte **1**) or for the protons directly on the methylene bridge (for analyte **2**), which indicates the existence of significant non-covalent interactions between this part of the molecule and the cyclodextrin host (Yang et al., 2008). Such interactions are likely intermolecular hydrogen bonding between the hydroxyls located at the cyclodextrin rim and the protons between the aromatic rings, which in turn activates the benzylic position for the desired oxidation reactions.

**Table 3:** Changes in the ^1^H NMR spectral signals of protons on bisphenol A as a function of added equivalents of α-cyclodextrin (α-CD)^a^.

**Eq. of α-CD**	***Methyl* protons (Δppm)**	***Ortho* protons (Δppm)^b^**	***Meta* protons (Δppm)^c^**
0.5	0.0291	0.0044	0.0609
1.0	0.0400	0.0071	0.0801
1.5	0.0443	0.0077	0.0870
2.0	0.0493	0.0089	0.0920
3.0	0.0522	0.0088	0.0953
5.0	0.0609	0.0135	0.1015

a *Δppm is defined as the difference in chemical shifts in the presence of cyclodextrin compared to the chemical shifts in the absence of cyclodextrin, according to the following equation*.

*Δppm = δ_complex_ (chemical shifts in presence of α-CD) – δ_control_ (chemical shifts without α-CD)*.

b *Ortho protons are defined as the protons that are at the ortho positions of the aromatic ring relative to the non-aromatic bridge*.

c *Meta protons are defined as the protons that are at the meta positions of the aromatic ring relative to the non-aromatic bridge*.

Of note, binding of aromatic compounds in cyclodextrin that leads to activation of benzylic positions through association with the cyclodextrin rim is a phenomenon that has been reported previously in the literature, both by our group (Chaudhuri et al., 2016) and by others (Andres and de Rossi, 2003; Lopez et al., 2007). In particular, a previous report by our group uses binding of aromatic rings in the cyclodextrin cavity to activate the benzylic position of benzylic alcohols and achieve effective and mild oxidation to the corresponding aldehydes (den Braver-Sewradj et al., 2020). Similarly, complexation in α-cyclodextrin, reported herein, has the dual function of protecting the aromatic ring from undesired chlorination and of facilitating effective oxidation at the benzylic site.

### Practical Applications

Real-world municipal waste water effluent samples, prior to chlorination, were used to simulate this mediated oxidation in practice. Such samples were doped with 100 mg/L of BPA (analyte **1**), and then treated with chlorine dioxide in the presence or absence of α-cyclodextrin. Results of these studies showed that α-cyclodextrin promoted the decomposition of BPA to form hydroquinone and chlorohydroquinone, with substantially more of these products formed in the presence of α-cyclodextrin compared to the decomposition run in the absence of cyclodextrin (Table 4). Nonetheless, the ratio of hydroquinone to chlorohydroquinone remained roughly unchanged by the addition of α-cyclodextrin, a result which is surprising based on the documented ability of α-cyclodextrin to affect this product distribution (*vide supra*). Reasons for this anomalous behavior may relate to the presence of interfering species in the wastewater sample. In particular, higher ionic strength and/or other species complexing with cyclodextrin sites can limit the ability to target specific compounds for specific oxidation mechanisms. Such treatment might be better suited in the long-term for industrial wastewater streams where a more consistent water matrix and a higher concentration of the targeted pollutant is available for cyclodextrin-mediated oxidation. We expect that additional modifications to our cyclodextrin-based system will allow for improved performance in such samples, possibly through combining the α-cyclodextrin with other additives that will address the more complex nature of real-world aqueous samples.

**Table 4:** Summary of the formation of hydroquinone and chlorohydroquinone from chlorine dioxide mediated decomposition of BPA in real-world wastewater samples, measured as normalized integrated peak emissions from GC-MS.

**Decomposition product**	**Without α-CD**	**With α-CD**
Hydroquinone^a^	0.17	0.31
Chlorohydroquinone^a^	0.51	1.00
Ratio of hydroquinone to chlorohydroquinone^b^	0.33	0.31

a *Values reported herein represent the integrated area of the GC-MS peaks that correspond to each analyte, with the results normalized so that the highest value peak (chlorohydroquinone in the presence of α-CD) is equal to 1.0*.

b *Ratio of hydroquinone to chlorohydroquinone is calculated as the quotient of the integrated area of the peak corresponding to hydroquinone divided by the integrated area of the peak corresponding to chlorohydroquinone*.

## Conclusions

The ability to change the product distribution of chlorine dioxide mediated degradation of organic pollutants via supramolecular complexation of the pollutants has substantial practical benefit in improving wastewater treatment methodologies, and is of interest from a fundamental scientific perspective as well. Results reported herein highlight that the use of α-cyclodextrin to bind small aromatic pollutants affects the accessibility of the structure to the chlorine dioxide reagent and the resulting distribution of oxidation to chlorination products in a way that is highly dependent on the structure of the reagent, the temperature of the treatment, and the molar equivalents of α-cyclodextrin added, with most cases resulting in a marked decrease in the relative amounts of chlorinated byproducts obtained. Reasons for these effects rely on the supramolecular complexation of the pollutants, confirmed by ^1^H NMR analysis, which cause steric shielding of the phenyl groups of the pollutants to undesired chlorination. Overall, the addition of α-cyclodextrin generally increases the ratio of water-soluble oxidation products and decreases the amount of toxic chlorination products, through the addition of a non-toxic, sugar-based cyclodextrin additive. The results reported herein provide significant groundwork for further development of novel and highly effective water treatment procedures, and open the possibility of using cyclodextrin-mediated complexation in other water treatment and pollutant removal processes.

## Data Availability Statement

All datasets generated for this study are included in the article/Supplementary Material.

## Author Contributions

SC ran the initial experiments on purified laboratory systems, identified system components, did 1H NMR analysis, and drafted the initial manuscript. DD ran experiments on real-world wastewater samples, finalized experiments that were left from SC, and edited the manuscript. TB provided intellectual insight throughout the project and edited the manuscript. AB provided industrial perspective on the project, as well as real-world wastewater samples, and also edited the manuscript. ML provided intellectual insight on the project, worked with SC to draft the initial manuscript, and took responsibility for all edits and corrections to arrive at this final version. All authors contributed to the article and approved the submitted version.

## Conflict of Interest

AB was employed by the company International Dioxcide. The remaining authors declare that the research was conducted in the absence of any commercial or financial relationships that could be construed as a potential conflict of interest. The authors declare that this study received funding from International Dioxcide. The funder was not involved in the study design, collection, analysis, interpretation of data, the writing of this article, or the decision to submit it for publication.
