# Supplementary material for: Use of α-cyclodextrin to Promote Clean and Environmentally Friendly Disinfection of Phenolic Substrates via Chlorine Dioxide Treatment
Source: Front Chem. 2020 Jul 31;8:641. doi: 10.3389/fchem.2020.00641 (PMC7413072; doi:10.3389/fchem.2020.00641)
Supplement: Supplementary file 1 [file Data_Sheet_1.PDF]

Electronic Supporting Information for

Use of α-cyclodextrin to promote clean and environmentally friendly disinfection of phenolic substrates via chlorine dioxide treatment

Sauradip Chaudhuri, Dana J. DiScenza, Thomas B. Boving, Alan Burke, and Mindy Levine

TABLE OF CONTENTS

Materials and Methods……………………………………………………………………………….S3

Detailed Experimental Procedures…………………………………………………………………...S4

Experimental Procedure for the Preparation of Chlorine Dioxide Solution……………………….S4

Experimental Procedure for the Complexation of Chlorine Dioxide with α-Cyclodextrin………S4

Experimental Procedure for the Determination of the Reaction Progression……………………...S4

Experimental Procedure for Measuring the Binding of Analytes in α-Cyclodextrin……………...S4

Experimental Procedure for the Treatment of Wastewater Effluent……………………………....S4

Summary Tables……………………………………………………………………………………..S5

Tables of ^{1}H NMR Results…………………………………………………………………..…S5

Tables of GC-MS Results……………………………………………………………………..…..S7

Summary Figures…………………………………………………………………………………...S15

Copies of ^{1}H NMR Spectra……………………………………………………………………....S15

Copies of GC-MS Chromatogram……………………………………………………………….S17

MATERIALS AND METHODS

^1H NMR experiments were conducted using a 400 MHz Bruker Avance spectrometer with D_2O as a solvent. GC-MS analyses of reaction mixtures were carried out using a Shimadzu GCMS-QP2020 instrument. All chemicals were purchased from Sigma Aldrich chemical company or from Fisher Scientific and were used as received, without further purification.

EXPERIMENTAL PROCEDURES

Experimental Procedure for the Preparation of Chlorine Dioxide Solution

An aqueous chlorine dioxide suspension was generated from the treatment of a solution of $NaClO_2$ (ADOX^{TM} 7.5) with activated HCl. A typical generation procedure involved the addition of 4 mL of 30-36% HCl to a mixture of 17.5 mL of ADOX^{TM} 7.5 and 200 mL of deionized water. The reaction mixture was kept in a dark Amber bottle for approximately 24 hours at room temperature. Prior to usage, the reaction mixture was further diluted to render a final chlorine dioxide concentration of 1095 ppm, with the final concentration determined using a pocket digital hand-held colorimeter

Experimental Procedure for the Complexation of Chlorine Dioxide with α-Cyclodextrin

The complexation of chlorine dioxide with α-cyclodextrin was obtained by mixing a solution of 6.2 mL of 1100 ppm of ClO_2 (100 μmol) with solid α-cyclodextrin (584 mg; 600 μmol) for 15-20 mins.

Experimental Procedure for the Determination of the Reaction Progression

The reaction mixture containing the organic analyte was treated with ClO_2 and allowed to react at the specified temperature for a certain amount of time, after which time the mixture was treated with concentrated sodium sulfite (Na_2SO_3) solution to quench the excess chlorine dioxide. The resulting solution was extracted with ethyl acetate. An aliquot of the organic phase was injected into the GC-MS for analysis. The unreacted starting material (analyte) was identified and the new product peaks were determined.

Experimental Procedure for Measuring the Binding of Analytes in α-Cyclodextrin

Binding of analytes with α-cyclodextrin was investigated via ^{1}H NMR titrations.[i] A mixture of analytes (20 μmol) with α-cyclodextrin (0.0 - 5.0 equivalents) in D_2O were investigated via ^{1}H NMR, and the resulting shifts in the positions of the NMR signals were fitted to mathematical equations to identify the best mathematical fit and determine the binding constant of the host-guest complex.

Experimental Procedure for the Treatment of Wastewater Effluent

Real-world wastewater samples were obtained from a Quonset, Rhode Island water treatment plant and stored in the refrigerator until analysis. Samples were treated in five different ways: through the use of ClO_2, ClO_2 and cyclodextrin, bleach, solid ClO_2 complex, and a control sample with no treatment.

Samples were transferred to a clean separatory funnel, extracted with ethyl acetate, and an aliquot of the organic layer was transferred to a glass vial for analysis via GC-MS.

SUMMARY TABLES

Summary Tables of ^{1}H NMR Results

Bisphenol A

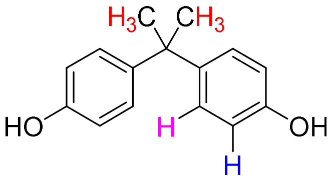

Table S1. Changes in the ^{1}H NMR spectral signals of protons on bisphenol A as a function of added equivalents of α-cyclodextrin (α-CD)[a]

Equivalents of α-CD	***Methyl* protons (red) (Δ ppm)**	***Ortho* protons (magenta) (Δ ppm)[b]**	***Meta* protons (blue) (Δ ppm)[c]**
0.5	0.0291	0.0044	0.0609
1.0	0.0400	0.0071	0.0801
1.5	0.0443	0.0077	0.0870
2.0	0.0493	0.0089	0.0920
3.0	0.0522	0.0088	0.0953
5.0	0.0609	0.0135	0.1015

[a] Δ ppm is defined as the difference in chemical shifts in the presence of cyclodextrin compared to the chemical shifts in the absence of cyclodextrin, according to the following equation:

Δ ppm = $\delta_{complex}$ (chemical shifts in presence of α-CD) - $\delta_{control}$ (chemical shifts without α-CD)

[b] *Ortho* protons are defined as the protons that are at the *ortho* positions of the aromatic ring relative to the non-aromatic bridge

[c] *Meta* protons are defined as the protons that are at the *meta* positions of the aromatic ring relative to the non-aromatic bridge

Bisphenol F

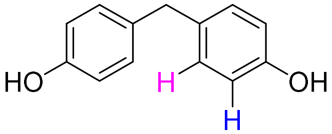

Table S2. Changes in the ^{1}H NMR spectral signals of protons on bisphenol F as a function of added equivalents of α-cyclodextrin (α-CD)[a]

Equivalents of α-CD	***Ortho* protons (magenta) (Δ ppm)[b]**	***Meta* protons (blue) (Δ ppm)[c]**
0.5	0.0098	0.0295
1.0	0.0171	0.0485
1.5	0.0228	0.0624
2.0	0.0281	0.0725
3.0	0.0376	0.0898
5.0	0.0521	0.1140

[a] Δppm is defined as the difference in chemical shifts in the presence of cyclodextrin compared to the chemical shifts in the absence of cyclodextrin, according to the following equation:

$\Delta ppm = \delta_{complex}$ (chemical shifts in presence of α-CD) - $\delta_{control}$ (chemical shifts without α-CD)

[b] *Ortho* protons are defined as the protons that are at the *ortho* positions of the aromatic ring relative to the non-aromatic bridge

[c] *Meta* protons are defined as the protons that are at the *meta* positions of the aromatic ring relative to the non-aromatic bridge

2-Phenylphenol

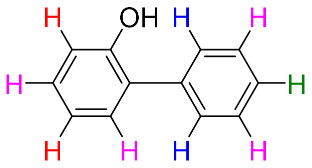

Table S3. Changes in the ^{1}H NMR spectral signals of protons on 2-phenylphenol as a function of added equivalents of α-cyclodextrin (α-CD)[a]

Equivalents. of α-CD	Red protons (Δ ppm)	Blue protons (Δ ppm)	Green proton (Δ ppm)	Magenta protons (Δ ppm)
0.5	0.0196	-0.0114	0.0077	0.0189
1.0	0.0416	-0.0040	0.0171	0.0335
1.5	0.0592	0.0028	0.0262	0.0443
2.0	0.0740	0.0078	0.0332	0.0510
3.0	0.0884	0.0172	0.0577	0.0685
5.0	0.1364	0.0311	0.0713	0.0850

[a] Δppm is defined as the difference in chemical shifts in the presence of cyclodextrin compared to the chemical shifts in the absence of cyclodextrin, according to the following equation:

$\Delta ppm = \delta_{complex}$ (chemical shifts in presence of α-CD) - $\delta_{control}$ (chemical shifts without α-CD)

Summary Tables of GC-MS Results

The GC-MS results varied significantly depending on the identity of the analyte investigated

*BISPHENOL A (ANALYTE **1**)*

Table S4. Results obtained from GC-MS analysis of the treatment of bisphenol A with chlorine dioxide in the presence of 0 equivalents of α-cyclodextrin[a] at 40°C for 24 hours

Retention Time (min)	Area %	Compound Category/ Identity
4.53	14.4	Higher mass byproduct (m/z = 281)
5.77	16.7	Chloroquinone
6.12	20.7	Higher mass byproduct (m/z = 355)
6.49	1.8	Methylquinone
7.77	5.2	Higher mass byproduct (m/z = 429)
8.19	35.7	Chloroquinol
9.89	1.7	Chloroethylquinone
10.27	3.8	Dichloroquinol

[a] The percent area values were calculated by integrating the peak areas of all peaks with greater than 1% relative area, and calculating the ratio of peak area of a particular peak to the overall peak areas determined.

Table S5. Results obtained from GC-MS analysis of the treatment of bisphenol A with chlorine dioxide in the presence of 27.4 equivalents of α-cyclodextrin at 40°C for 24 hours

Retention Time (min)	Area %	Compound Category/ Identity
4.56	11.9	Higher mass byproduct (m/z = 281)
5.78	13.1	Chloroquinone
6.13	20.3	Higher mass byproduct (m/z = 355)
7.78	9.3	Higher mass byproduct (m/z = 429)
8.19	41.0	Chloroquinol
10.27	4.6	Dichloroquinol

[a] The percent area values were calculated by integrating the peak areas of all peaks with greater than 2% relative area, and calculating the ratio of peak area of a particular peak to the overall peak areas determined.

Table S6. Results obtained from GC-MS analysis of the treatment of bisphenol A with chlorine dioxide in the presence of 68.5 equivalents of α-cyclodextrin at 40ºC for 24 hours

Retention Time (min)	Area %	Compound Category/ Identity
4.58	11.7	Higher mass byproduct (m/z = 281)
5.81	10.6	Chloroquinone
6.15	22.1	Higher mass byproduct (m/z = 355)
7.78	7.8	Higher mass byproduct (m/z = 429)
8.20	45.5	Chloroquinol
9.27	2.1	Higher mass byproduct (m/z = 503)

[a] The percent area values were calculated by integrating the peak areas of all peaks with greater than 2% relative area, and calculating the ratio of peak area of a particular peak to the overall peak areas determined.

Table S7. Results obtained from GC-MS analysis of the treatment of bisphenol A with chlorine dioxide in the presence of 0 equivalents of α-cyclodextrin at room temperature for 24 hours, with significant unreacted starting material observed at 14.58 minutes

Retention Time (min)	Area %	Compound Category/Identity
5.79	4.3	Byproduct
6.13	4.0	Byproduct
7.78	2.4	Oxidation product (quinols/quinones)
8.19	11.6	Oxidation product (quinols/quinones)
10.27	3.7	Byproduct
14.58	71.4	Leftover starting material
14.99	2.7	Chlorinated starting material

[a] The percent area values were calculated by integrating the peak areas of all peaks with greater than 2% relative area, and calculating the ratio of peak area of a particular peak to the overall peak areas determined.

Table S8. Results obtained from GC-MS analysis of the treatment of bisphenol A with chlorine dioxide in the presence of 27.4 equivalents of α-cyclodextrin at room temperature for 24 hours, with significant unreacted starting material observed at 14.54 minutes

Retention Time (min)	Area %	Compound Category/Identity
5.77	5.1	Byproduct
6.12	5.6	Byproduct
7.54	2.3	Oxidation product (quinols/quinones)
7.78	3.1	Oxidation product (quinols/quinones)
8.19	26.1	Oxidation product (quinols/quinones)
10.28	2.8	Byproduct
14.54	53.2	Leftover starting material
14.79	0.6	Chlorinated starting material
14.99	1.4	Chlorinated starting material

[a] The percent area values were calculated by integrating the peak areas of all peaks with greater than 0.5% relative area, and calculating the ratio of peak area of a particular peak to the overall peak areas determined.

Table S9. Results obtained from GC-MS analysis of the treatment of bisphenol A with chlorine dioxide in the presence of 68.5 equivalents of α-cyclodextrin at room temperature for 24 hours, showing significant unreacted starting material at 14.56 minutes

Retention Time (min)	Area %	Compound Category/Identity
5.67	0.7	Byproduct
5.81	1.2	Byproduct
6.14	3.6	Byproduct
6.87	1.9	Byproduct
7.55	5.4	Oxidation product (quinols/quinones)
7.78	1.5	Oxidation product (quinols/quinones)
8.20	19.5	Oxidation product (quinols/quinones)
9.27	0.6	Byproduct
14.56	65.0	Leftover starting material
14.99	0.5	Chlorinated starting material

[a] The percent area values were calculated by integrating the peak areas of all peaks with greater than 0.5% relative area, and calculating the ratio of peak area of a particular peak to the overall peak areas determined.

Table S10. Ratio of oxidation to chlorination products formed from the chlorine dioxide-mediated decomposition of bisphenol A at room temperature after 24 hours

Eq. of α-CD	Oxidation product %	Chlorination product %	Unreacted starting material %
0.0 eq.	13.0%	2.7%	71.4%
27.4 eq.	31.5%	1.9%	53.2%
68.5 eq.	26.4%	0.5%	65.0%

Table S11. Ratio of chloroquinol to chloroquinone formed from the chlorine dioxide-mediated decomposition of bisphenol A at 40 ºC after 24 hours

**Eq. of α-CD**	**Chloroquinol/Chloroquinone Ratio**	**Overall oxidation product %**
0	1.9	56.2%
27.4	3.5	53.6%
68.5	4.3	55.3%

*BISPHENOL F (ANALYTE **2**)*

Table S12. Results obtained from GC-MS analysis of the treatment of bisphenol F with chlorine dioxide in the presence of 0 equivalents of α-cyclodextrin at 40ºC for 24 hours, showing 100% oxidation to quinones, with some amount of chlorination of the quinones also occurring

Retention Time (min)	Area %	Compound Category/ Identity
7.63	14.4	Oxidation products (quinols)
8.09	85.6	Oxidation + chlorination products (chloroquinols)

Table S13. Results obtained from GC-MS analysis of the treatment of bisphenol F with chlorine dioxide in the presence of 24 equivalents of α-cyclodextrin at 40ºC for 24 hours

Retention Time (min)	Area %	Compound Category/ Identity
7.56	33.7	Oxidation products (quinols)
8.07	66.3	Oxidation + chlorination products (chloroquinols)

Table S14. Results obtained from GC-MS analysis of the treatment of bisphenol F with chlorine dioxide in the presence of 60 equivalents of α-cyclodextrin at 40ºC for 24 hours

Retention Time (min)	Area %	Compound Category/ Identity
7.62	34.6	Oxidation products (quinols)
8.07	65.4	Oxidation + chlorination products (chloroquinols)

Table S15. Ratio of oxidation to chlorination products obtained from chlorine dioxide mediated decomposition of bisphenol F at 40 ºC for 24 hours[a]

Equivalents of α-CD	Ratio of oxidation to chlorination products
0	1.2
24	1.5
60	1.5

[a] All reactions yielded 100% oxidation to quinones, and some percentage of those were chlorinated as well, so this value was calculated as 100% divided by the percentage of the quinone that was also chlorinated

*2-PHENYLPHENOL (ANALYTE **3**)*

Table S16. Results obtained from GC-MS analysis of the treatment of 2-phenylphenol with chlorine dioxide in the presence of 0 equivalents of α-cyclodextrin at 40ºC for 24 hours

Retention Time (min)	Area %	Compound Category/ Identity
4.50	1.9	Byproduct
6.12	5.2	Byproduct
6.79	25.2	Oxidation products (quinones)
7.77	2.9	Byproduct
8.40	1.2	Byproduct
9.03	0.8	Byproduct
10.00	2.0	Byproduct
10.72	16.1	Oxidation products (quinones)
11.98	2.0	Byproduct
12.58	16.6	Oxidation products (quinols)
13.80	15.0	Oxidation + chlorination products (chloroquinones)
14.08	11.1	Oxidation + chlorination products (chloroquinols)

[a] The percent area values were calculated by integrating the peak areas of all peaks with greater than 0.5% relative area, and calculating the ratio of peak area of a particular peak to the overall peak areas determined.

Table S17. Results obtained from GC-MS analysis of the treatment of 2-phenylphenol with chlorine dioxide in the presence of 20.6 equivalents of α-cyclodextrin at 40°C for 24 hours

Retention Time (min)	Area %	Compound Category/ Identity
4.58	3.0	Byproduct
6.15	7.0	Byproduct
6.76	28.9	Oxidation products (quinones)
7.78	4.6	Byproduct
8.41	1.0	Byproduct
9.27	1.3	Byproduct
10.00	2.1	Byproduct
10.71	14.2	Oxidation products (quinones)
12.38	10.1	Oxidation products (quinols)
12.57	9.9	Oxidation products (quinols)
13.79	15.3	Oxidation + chlorination products (chloroquinones)
14.09	2.7	Oxidation + chlorination products (chloroquinols)

[a] The percent area values were calculated by integrating the peak areas of all peaks with greater than 0.5% relative area, and calculating the ratio of peak area of a particular peak to the overall peak areas determined.

Table S18. Results obtained from GC-MS analysis of the treatment of 2-phenylphenol with chlorine dioxide in the presence of 51.5 equivalents of α-cyclodextrin at 40°C for 24 hours

Retention Time (min)	Area %	Compound Category/ Identity
4.50	2.6	Byproduct
6.12	6.9	Byproduct
6.75	23.0	Oxidation products (quinones)
7.77	3.2	Byproduct
9.27	0.9	Byproduct
10.00	2.2	Byproduct
10.72	16.9	Oxidation products (quinones)
12.42	27.7	Oxidation products (quinols)
13.80	16.6	Oxidation + chlorination products (chloroquinones)

[a] The percent area values were calculated by integrating the peak areas of all peaks with greater than 0.5% relative area, and calculating the ratio of peak area of a particular peak to the overall peak areas determined.

Table S19. Summary of oxidation to chlorination ratios obtained from the chlorine dioxide mediated decomposition of 2-phenylphenol at 40 ºC for 24 hours[a]

Eq. of α-CD	Oxidation products to quinones	Oxidation products to quinols	Oxidation and chlorination to chloroquinols/ones	Ratio of oxidation to chlorination	Byproduct formation %
0	41.3%	16.6%	26.1%	3.2	16.2%
20.6	43.1%	20.0%	18.0%	4.5	18.9%
51.5	39.9%	27.7%	16.6%	5.1	15.8%

[a] Results demonstrated significant oxidation to quinones, and of the quinones, many of them were also chlorinated to form chloroquinones. Ratios were determined by calculating the amount of quinone formation (including chloroquinones) divided by the amount of chloroquinone formation

SUMMARY FIGURES

Copies of ^{1}H NMR Spectra

Bisphenol A

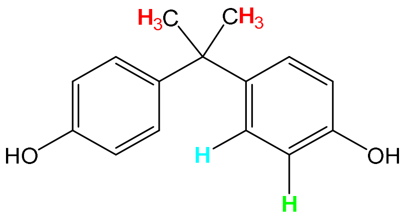

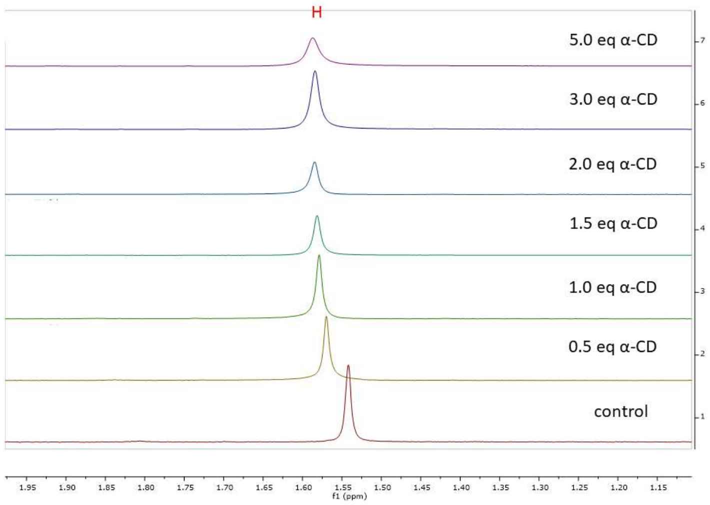

Figure S1. ^{1}H NMR spectrum of bisphenol A in the presence of increasing equivalents of a-cyclodextrin, focused on the aliphatic proton spectral region

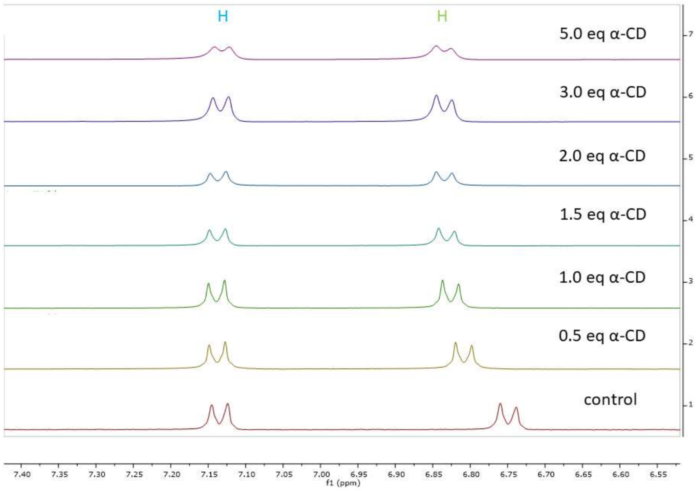

Figure S2. ^{1}H NMR spectrum of bisphenol A in the presence of increasing equivalents of a-cyclodextrin, focused on the aromatic proton spectral region

Bisphenol F

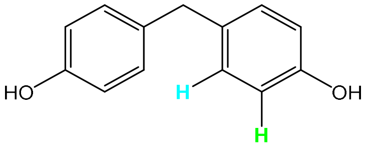

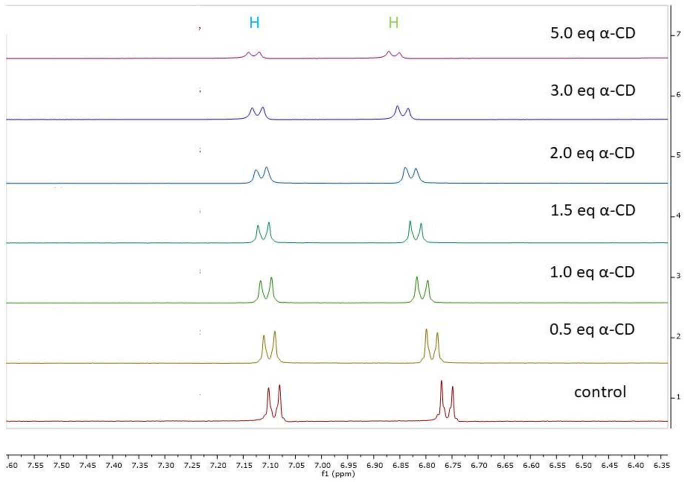

Figure S3. ^{1}H NMR spectrum of bisphenol F in the presence of increasing equivalents of a-cyclodextrin, focused on the aromatic proton spectral region

2-Phenylphenol

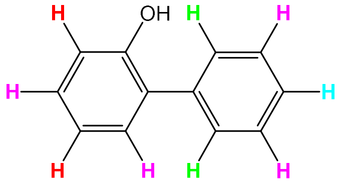

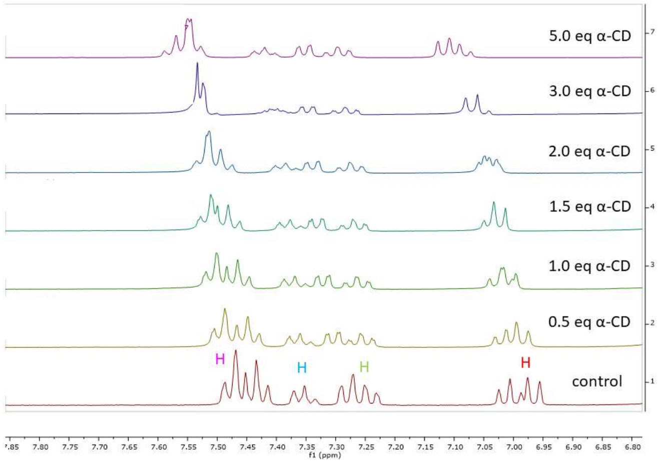

Figure S4. ^{1}H NMR spectrum of 2-phenylphenol in the presence of increasing equivalents of a-cyclodextrin, focused on the aromatic proton spectral region

Copies of GC-MS Spectra

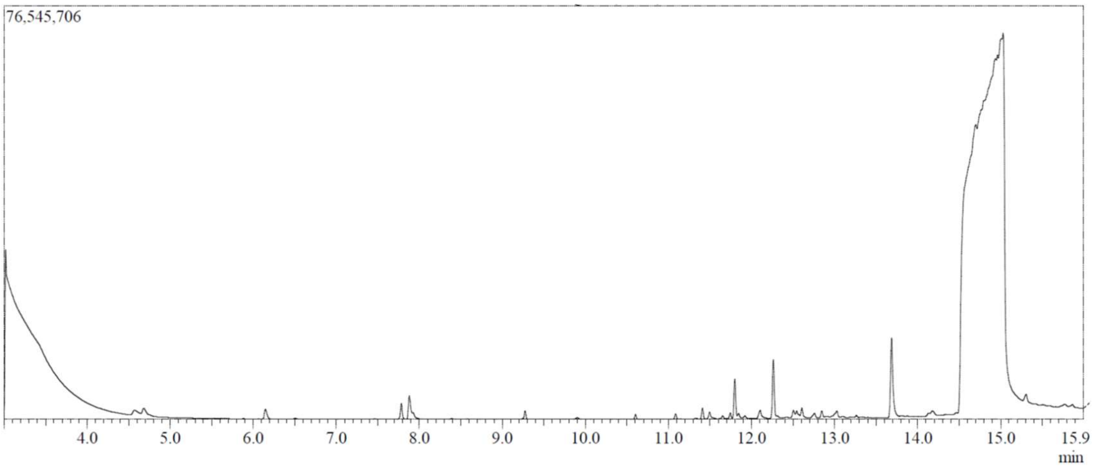

Figure S5. Bisphenol A

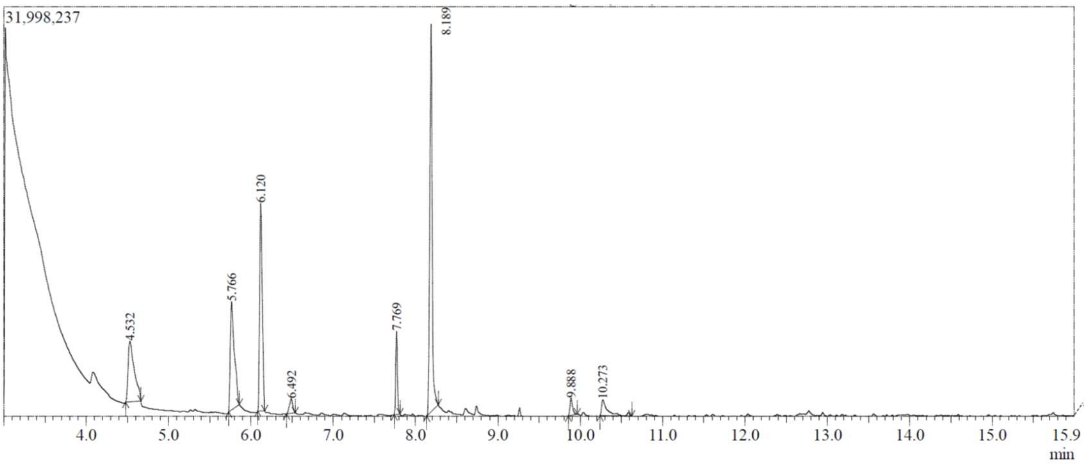

Figure S6. GC-MS chromatogram from the treatment of bisphenol A with chlorine dioxide at 40 °C with 0 equivalents of α-cyclodextrin

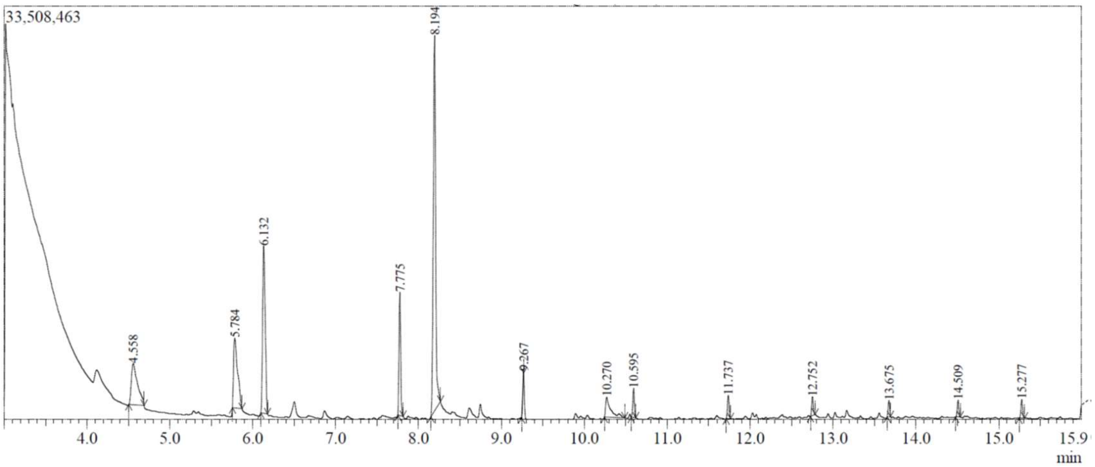

Figure S7. GC-MS chromatogram from the treatment of bisphenol A with chlorine dioxide at 40 °C with 27.4 equivalents of α-cyclodextrin

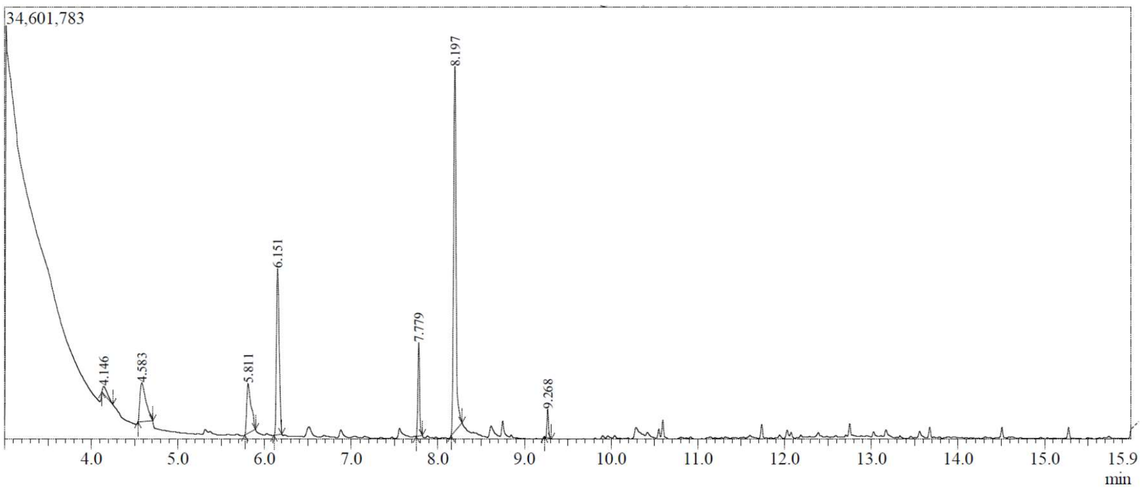

Figure S8. GC-MS chromatogram from the treatment of bisphenol A with chlorine dioxide at 40 °C with 68.5 equivalents of α-cyclodextrin

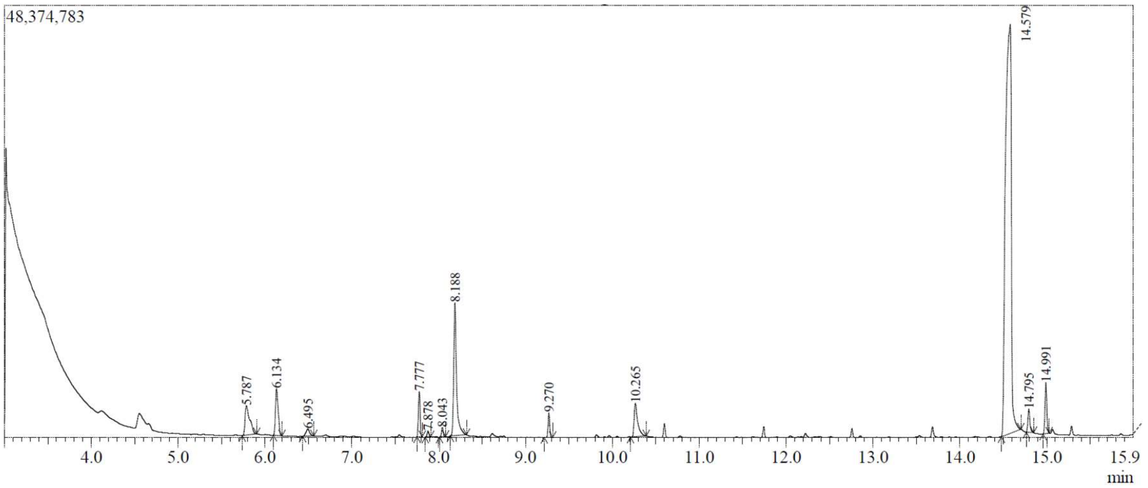

Figure S9. GC-MS chromatogram from the treatment of bisphenol A with chlorine dioxide at room temperature with 0 equivalents of α-cyclodextrin

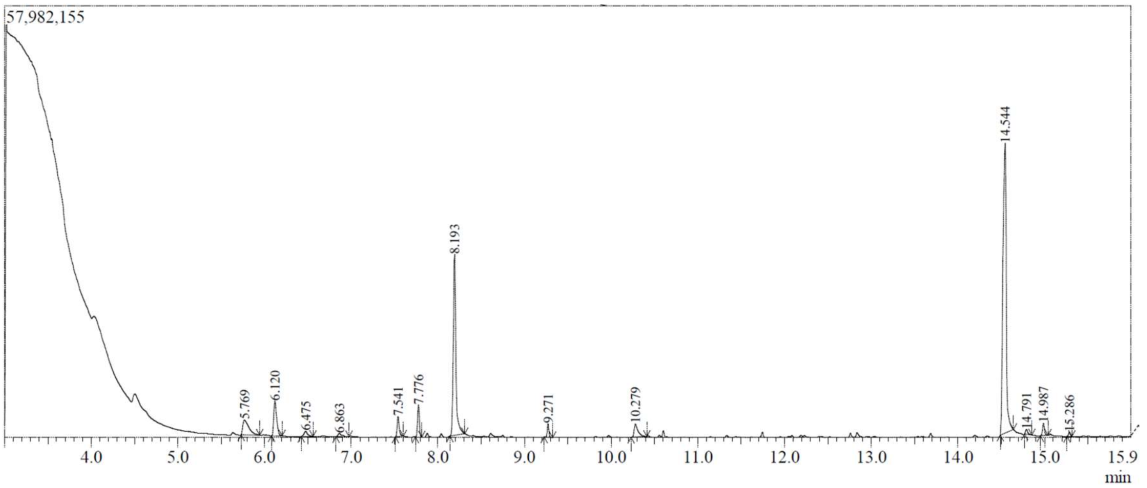

Figure S10. GC-MS spectrum from the treatment of bisphenol A with chlorine dioxide at room temperature with 27.4 equivalents of α-cyclodextrin

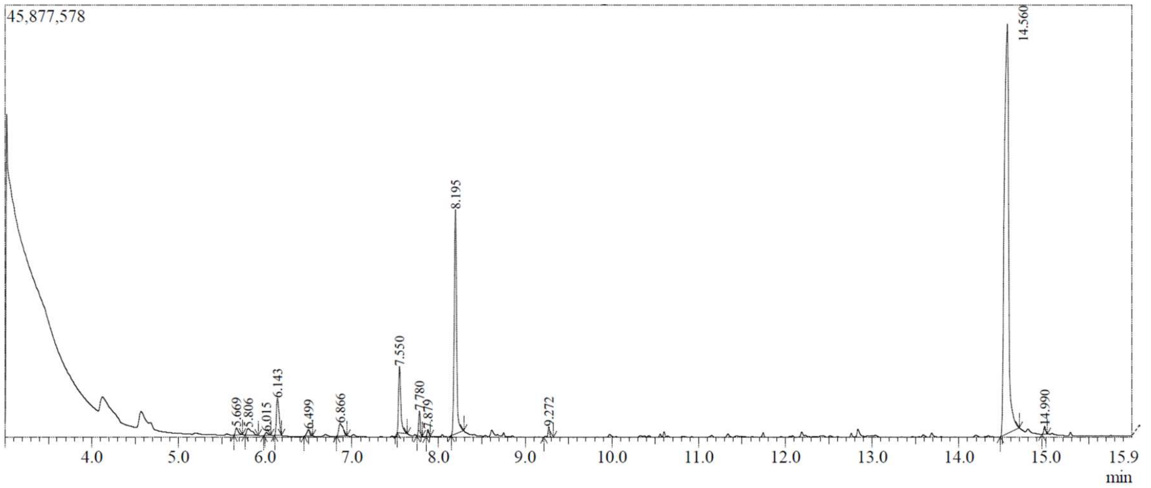

Figure S11. GC-MS spectrum from the treatment of bisphenol A with chlorine dioxide at room temperature with 68.5 equivalents of α-cyclodextrin

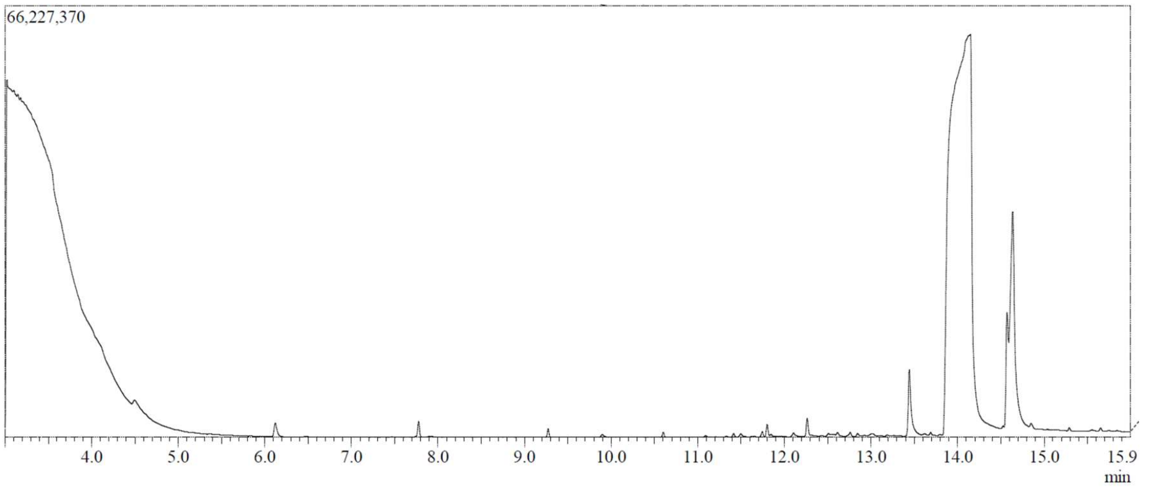

Figure S12. GC-MS spectrum of bisphenol F

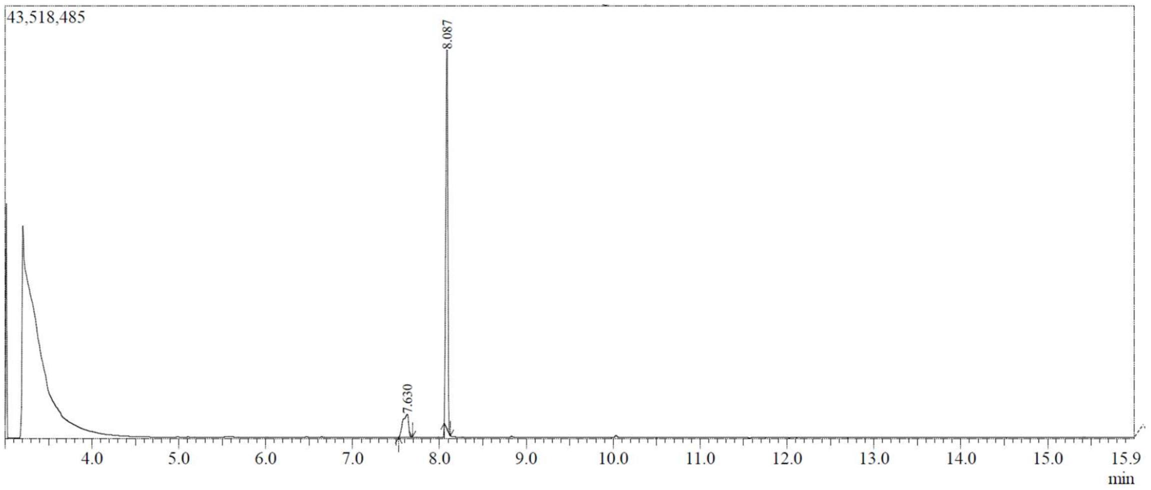

Figure S13. GC-MS spectrum from the treatment of bisphenol F with chlorine dioxide at 40 °C with 0 equivalents of α-cyclodextrin

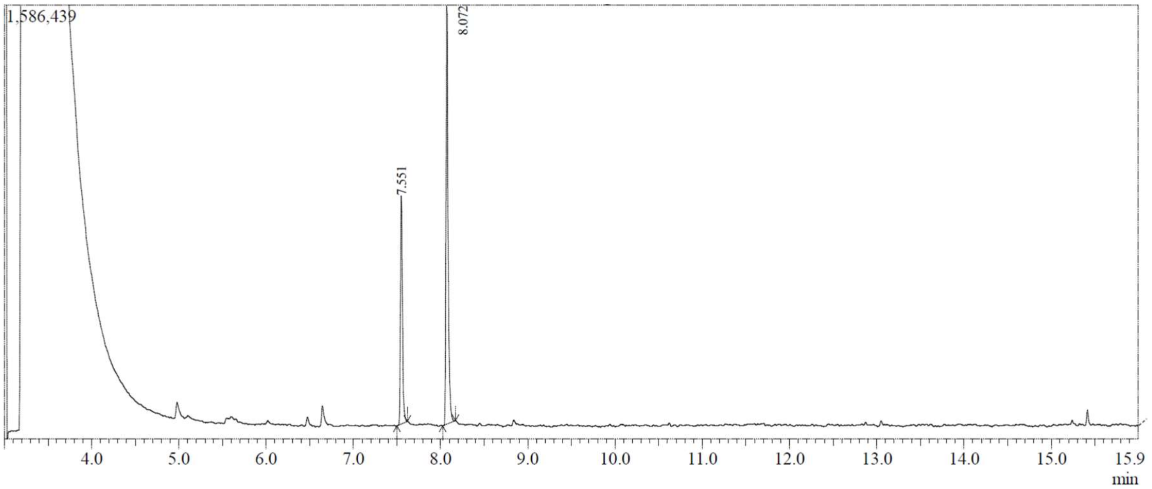

Figure S14. GC-MS spectrum from the treatment of bisphenol F with chlorine dioxide at 40 °C with 24 equivalents of α-cyclodextrin

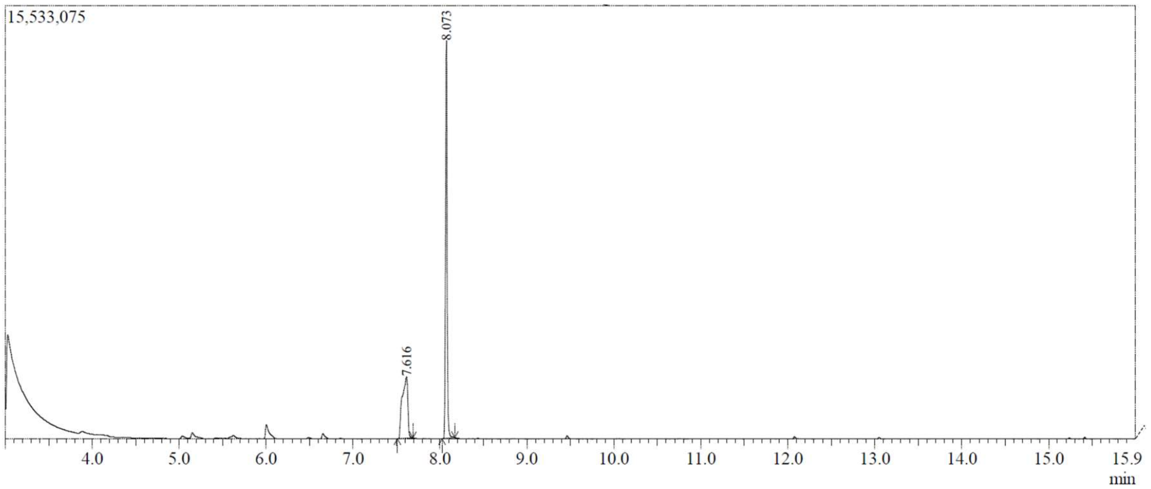

Figure S15. GC-MS spectrum from the treatment of bisphenol F with chlorine dioxide at 40 °C with 60 equivalents of α-cyclodextrin

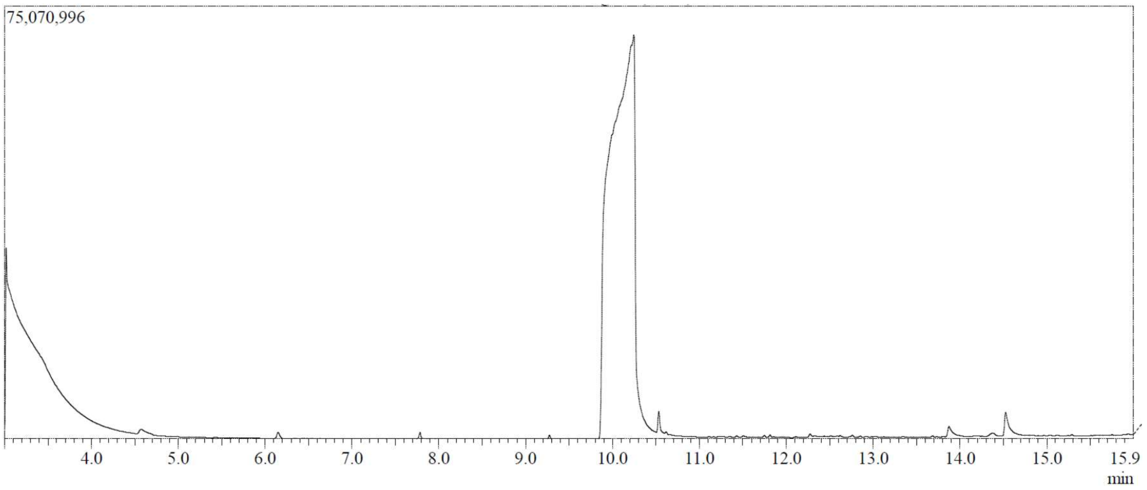

Figure S16. GC-MS spectrum of 2-phenylphenol.

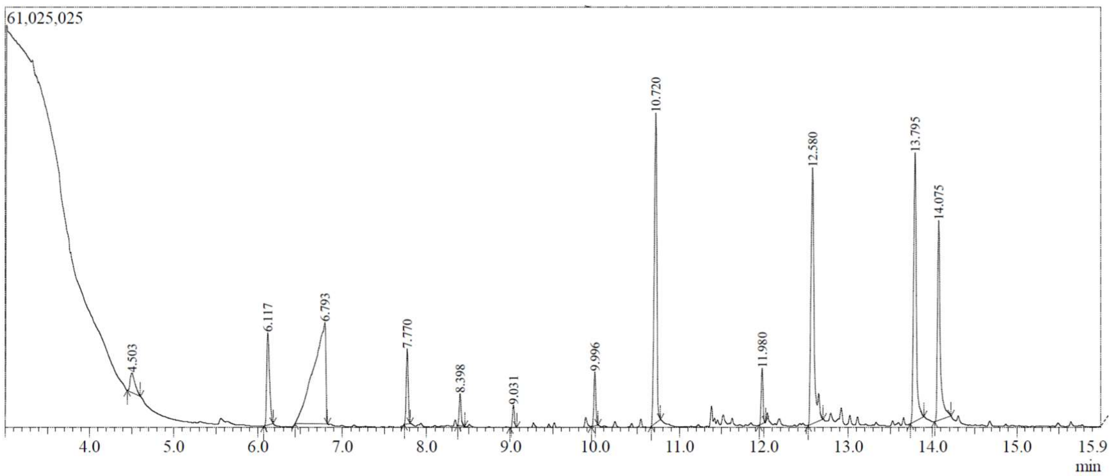

Figure S17. GC-MS spectrum from the treatment of 2-phenylphenol with chlorine dioxide at 40 °C with 0 equivalents of α-cyclodextrin

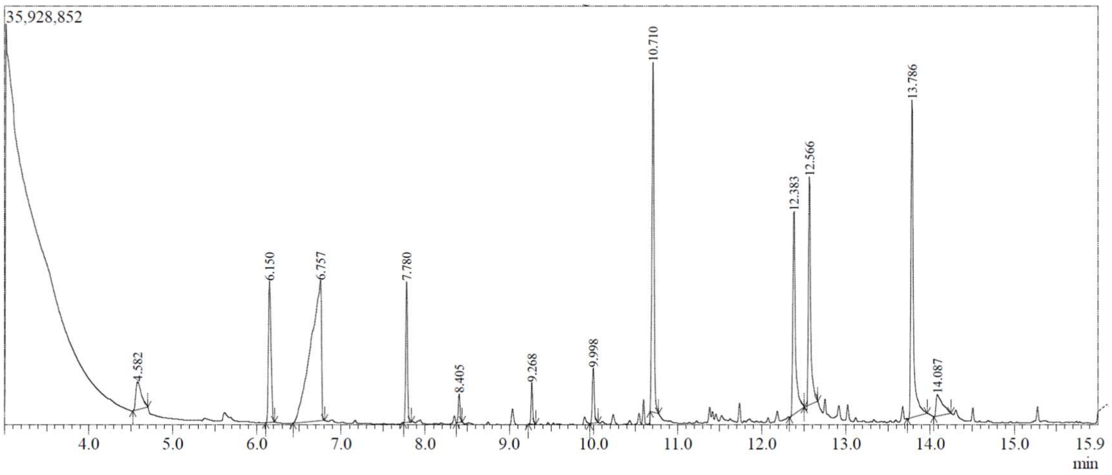

Figure S18. GC-MS spectrum from the treatment of 2-phenylphenol with chlorine dioxide at 40 °C with 20.6 equivalents of α-cyclodextrin

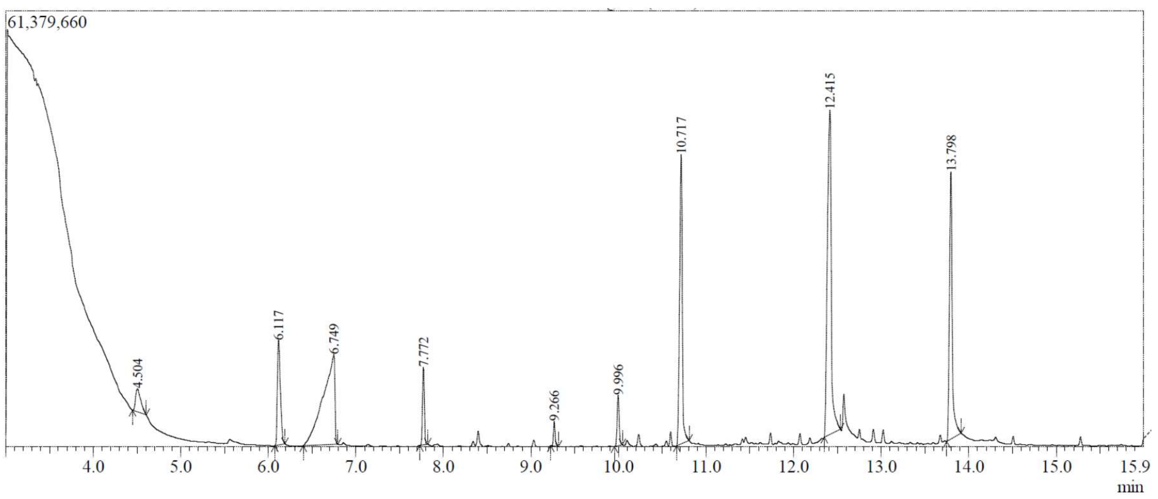

Figure S19. GC-MS spectrum from the treatment of 2-phenylphenol with chlorine dioxide at 40 °C with 51.5 equivalents of α-cyclodextrin

REFERENCES

---

[i] Roselet, S. Lizy; Kumari, J. P. Inclusion Studies on α-Cyclodextrin Complexes of Glipizide and Gliclazide with Effect of pH. *Asian J. Pharmaceutical Clinical Res.* **2017**, *10*, 273-280.
